# Change trajectory of fluid load management behavior ability in peritoneal dialysis patients and its association with physical activity

**DOI:** 10.3389/fmed.2025.1677026

**Published:** 2026-01-06

**Authors:** Yan Zheng, Jie Wang, Zhenzhen Chen, Lina Zhang, Fei Shang, Panpan Fu, Jinge Lian

**Affiliations:** 1 Department of Nephrology, Henan Provincial People's Hospital, Zhengzhou, China; 2 Department of Nephrology, Zhengzhou University People's Hospital, Zhengzhou, China; 3 Department of Nephrology, Henan Provincial Key Laboratory of Kidney Disease and Immunology, Zhengzhou, China; 4 Peritoneal Dialysis Center, Henan Provincial People's Hospital, Zhengzhou, China

**Keywords:** peritoneal dialysis, volume load, volume management, curves, trajectory, physical activity, longitudinal studies, influencing factors

## Abstract

**Objective:**

This study aims to explore the change trajectory of fluid load management ability in peritoneal dialysis (PD) patients and the correlation between different trajectories and physical activity.

**Methods:**

A total of 243 patients who underwent peritoneal dialysis were selected. A longitudinal investigation was carried out using the Peritoneal Dialysis Patient Volume Management Behavior Scale and the International Physical Activity Questionnaire-Long Form (IPAQ-LF).

**Results:**

Three trajectories of volume overload management behavioral ability were identified, namely C1 (low-level increasing group), C2 (medium-level increasing group), and C3 (low- to medium-level fluctuation group). There were significant differences between these categories in cultural-level trials (*χ*^2^ = 15.344, *p* = 0.018), diabetic nephropathy (*χ*^2^ = 11.267, *p* = 0.004), peritonitis during the study period (*χ*^2^ = 11.340, *p* = 0.003), and hypoalbuminemia (*χ*^2^ = 7.700, *p* = 0.021). During the first 6 months of initial peritoneal dialysis (T1–T4), each patient’s physical activity score increased [C1: (*F* = 107.250, *p* < 0.001); C2: (*F* = 45.383, p < 0.001); C3: (*F* = 30.194, *p* < 0.001)]. At the T1 stage, the physical activity score of group C2 was significantly higher than those of groups C1 and C3 (*p* < 0.01). At the T2 stage, the physical activity score of group C2 was significantly higher than that of group C3 (*p* < 0.001), and the physical activity score of group C1 was significantly higher than that of group C3 (*p* < 0.01). At the T3–T4 stage, the score of group C1 was significantly higher than that of groups C2 and C3 (*p* < 0.01), and the score of group C2 was significantly higher than that of group C3 (*p* < 0.001).

**Conclusion:**

Education level, diabetic nephropathy, concurrent peritonitis, and hypoproteinemia affect the change trajectory of volume load. Additionally, volume overload management at different stages influences the physical activity of patients.

## Introduction

Worldwide, there are approximately three million peritoneal dialysis (PD) patients, accounting for 11% of patients undergoing dialysis for end-stage renal disease, with an annual growth rate of 4–5% (1). In China, data show that there were approximately 120,000 peritoneal dialysis patients in 2023, with an annual growth rate of 8% (2).

Peritoneal dialysis (PD) has the advantages of autonomy, flexibility, home-based treatment, and preservation of residual renal function and has been adopted by the majority of patients with end-stage renal disease (3). With improvements in peritoneal dialysis technology, the mortality rate of patients has been greatly reduced (4). However, data show that there is still a high level of volume overload in 60% of patients. Higher levels of volume overload in patients are an important factor contributing to dialysis interruption, heart failure, and death (5, 6).

The conceptual framework of fluid load management behavior ability is a multidimensional structure based on the Transtheoretical Model of Behavior Change, encompassing four core components: stages of change, processes of change, self-efficacy, and decisional balance.

This ability specifically refers to the comprehensive skills and confidence that peritoneal dialysis patients possess to maintain fluid and electrolyte balance. It includes identifying their own volume status, adhering to treatment and dietary plans, conducting daily monitoring (e.g., of body weight and blood pressure), adjusting water and salt intake, and effectively coping with the physiological and psychological challenges encountered during the management process (7). Studies have indicated that patients with strong management abilities can control their water and salt intake more effectively, thereby reducing their volume load. This is manifested as more stable blood pressure and body weight, along with a lower risk of complications such as hypertension and heart failure (8).

Peritoneal dialysis patients with good volume-load management capacity can take specific actions based on their own physical condition, including limiting water and salt intake, choosing appropriate dialysis methods, and protecting peritoneal function. Therefore, high-level volume management behaviors of peritoneal dialysis patients are conducive to maintaining dialysis and are of great significance for long-term survival.

Studies have shown that the level of volume load has a significant influence on patients’ physical activity (9). Non-standard and poor physical activity in patients has a negative impact on their long-term prognosis and survival. Initial peritoneal dialysis patients may have a low level of volume management for several reasons, preventing them from transitioning to the maintenance phase of peritoneal dialysis (10, 11). Therefore, we propose the hypothesis that there may be significant differences in physical activity among peritoneal dialysis patients with volume management behaviors at different latent profile levels.

Therefore, identifying the change trajectory of volume management behavior ability in peritoneal dialysis patients, determining the development trend of volume management behavior ability, identifying high-risk groups that need intervention and management, and carrying out appropriate intervention and management are conducive to reducing the volume load level of patients and have a positive effect on their prognosis and development.

In view of this, this study used the latent class growth model (LCGM) to identify the change trajectory of volume overload management behavior in peritoneal dialysis patients, analyze the factors influencing different trajectories and their relationship with physical activity, and provide a theoretical basis for clinically recognizing the volume overload management behavior ability of high-risk groups and increasing physical activity.

## Subjects and methods

### Subjects

A total of 243 peritoneal dialysis patients admitted to Henan Provincial People’s Hospital were selected as study subjects using the convenience sampling method. This study was approved by the Ethics Committee of Henan Provincial People’s Hospital (No: Ethics 2021-Research-154).

#### Inclusion criteria

(1) Participants must meet the diagnostic criteria of chronic kidney disease and have a plan to receive peritoneal dialysis (12);
(2) They must be 18 years of age or older;
(3) Participants should provide informed consent for this study and participate voluntarily;
(4) They must have basic communication skills and have signed the informed consent.

#### Exclusion criteria

(1) Patients undergoing hemodialysis simultaneously.
(2) Patients with severe heart failure, malignant tumors, and other diseases.
(3) Patients who died or withdrew for other reasons.
(4) Those with missing questionnaires for more than one time.

### Research methods

#### Sample size requirements

This study was designed for longitudinal repeated measurement, and the sample size calculation formulas (13) $n=\frac{2}{\delta^{2}}[\sigma_{\mu }^{2}+\frac{1+(K-1)\rho_{c}}{K}{\sigma_{e}}^{2}]{\left(U_{\alpha /2}+U_{\beta }\right)}^{2}{\sigma_{e}}^{2}$, and five cases of small sample preliminary experiments resulted in ${\sigma_{e}}^{2}$=122.257, $\rho_{c}$=0.720, and $\sigma_{\mu }^{2}$=156.333, which resulted in n=161, the minimum sample size of 161 cases. Considering the loss rate of 10%, 161/0.90 = 179. This study included 243 cases.

#### Survey tool

(1) The basic information questionnaire consisted of demographic data of peritoneal dialysis patients, including sex, age, marital status, residence, education level, family per capita monthly income, types of chronic diseases, and comorbidities.
(2) The Volume Management Behavior Scale for Peritoneal Dialysis Patients was developed by Yi et al. (14). The scale had two dimensions, namely diet management and CAPD-related indicators, and a total of eight items. Each item was scored on a scale of 0–3 points. The total score of the scale ranged from 0 to 24, and the higher the score, the better the patient’s management behavior. The Cronbach’s *α* coefficients of the scale in this study were 0.824–0.840.
(3) International Physical Activity Questionnaire–Long Form (IPAQ-LF). The scale was prepared in Chinese by Fan et al. (15) and includes five dimensions and 27 items. Physical activity was assigned according to intensity, which was expressed as MET. The results were as follows: walking = 3.3, cycling = 6.0, moderate-intensity housework = 4.0, vigorous-intensity housework = 5.5, and vigorous-intensity physical activity at work and leisure = 8.0. The level of physical activity an individual engages in per week = MET value corresponding to that physical activity × duration of each activity (min) × number of times per week. A total physical activity level of <600 MET-min/week indicated a low level of physical activity, 600 ~ <3,000 MET-min/week indicated a moderate physical activity level, and >3,000 MET-min/week indicated a high physical activity level.

#### Questionnaire recovery and quality control

This study was conducted after obtaining informed consent from all participating patients, followed by approval from the hospital ethics committee. A longitudinal survey design was employed to collect data at four key time points relative to the peritoneal dialysis (PD) initiation: within the first week of initial dialysis (T1), at 1 month of regular dialysis (T2), at 3 months (T3), and at 6 months (T4). T1 questionnaires were administered during the patient’s hospital admission. At subsequent time points (T2–T4), peritoneal dialysis center staff conducted follow-ups to obtain data, ensuring that the assessments were conducted in the patients’ natural living environments and potentially enhancing the ecological validity of the responses regarding daily management behaviors.

All questionnaires, covering basic demographic information, capacity load management, behavioral capability, and physical activity levels, were administered anonymously to protect patient privacy. For patients with lower educational attainment or literacy challenges, the investigators implemented an adapted data collection procedure: the questionnaire content was presented orally in a standardized, neutral manner, and patients selected their answers from the provided options to minimize interviewer bias. This approach aimed to ensure that all participants, regardless of their cultural or educational background, could comprehend the questions and provide valid responses.

To ensure data quality and accuracy, the processes of data collection and entry were strictly separated and performed by two researchers who had received specialized training before the commencement of the study. One researcher was solely responsible for collecting the questionnaires and recording the initial responses. Subsequently, a second researcher, working independently, performed a thorough verification check of the original questionnaires before entering the data into the analysis database. This segregation of duties was designed to reduce the potential for data entry errors. Furthermore, to encourage continued participation and minimize attrition across the four time points, which is a common challenge in longitudinal studies, a small incentive gift was provided to patients who completed all questionnaires in the study.

### Statistical methods

IBM Statistical Package for the Social Sciences (SPSS) version 26.0 and Mplus 8.0 software were used for statistical analysis and data testing. The count data are presented as cases and percentages (%), and a chi-square analysis was performed. Measurement data are expressed as mean ± standard deviation (*x̄* ± *S*), and analysis of variance was applied for data statistics. For the first time, the intraclass variance was set at 0. Beginning with one model, the number of models was incrementally increased, and the optimal model was determined based on practical significance and fitting indices. The fitting indices encompassed the Akaike information criterion (AIC), Bayesian information criterion (BIC), and sample-corrected BIC (aBIC). Smaller statistical values signify better model fitting. Entropy represents the classification accuracy. Regarding the likelihood ratio test (LRT) and the bootstrap likelihood ratio test (BLRT), the principle of both is to compare the differences in model fitting between *k*-1 and *k* categories. The test level was set at *α* = 0.05.

## Results

### General demographic data

A total of 233 valid questionnaires were collected for this study. Among them, there were 135 males, accounting for 57.94%, and 98. Of this, 10 patients (42.06%) were lost to follow-up at the T4 stage (females, accounting for 42.06%). The average age was 56.85 ± 10.28 years (range: 32–84 years). Detailed information is shown in Table 1.

**Table 1:** Subjects of general information (*n* = 233).

Items	Categories	*N*	Percentage (%)
Age (years)	<45	49	21.03
	45–59	123	52.79
	≥60	61	26.18
Sex	Male	135	57.94
	Female	98	42.06
Occupations	Enterprises and institutions	22	9.44
	Farmer	38	16.31
	Staff/staff	90	38.63
	Retirement	46	19.74
	No regular occupation	37	15.88
Marital status	Married	206	88.41
	Unmarried	10	4.29
	Divorce	17	7.30
Place of residence	Rural	77	33.05
	Towns	156	66.95
Monthly income (yuan)	<2,000	39	16.74
	2,000–4,000	72	30.90
	4,001–6,000	77	33.05
	>6,000	45	19.31
Level of education	Primary school and below	46	19.74
	Junior high school	75	32.19
	Senior high school	69	29.61
	College and above	43	18.46
Hypertension	Yes	149	63.95
	No	84	36.05
Diabetic nephropathy	Yes	113	48.50
	No	120	51.50
Coronary heart disease	Yes	86	36.91
	No	147	63.09

### To determine the change trajectory of volume-load management behavior in peritoneal dialysis patients

In this study, 1–5 latent development trajectory models were constructed. Based on the results of the LRT and BLRT tests (*p* < 0.05), the 4- and 5-trajectory models were excluded. In the 1–3 trajectory models, by comparing the fitting indices, such as AIC, BIC, and aBIC, it was found that the values of each index showed a downward trend as the number of trajectory categories increased. However, priority should be given to maximizing entropy. After comprehensively evaluating the model fitness and classification accuracy, three trajectory categories were determined to be retained, as shown in Table 2.

**Table 2:** Comparison of analysis indicators of behavioral volume development trajectory types in peritoneal dialysis patients (*n* = 233).

Models	AIC	BIC	aBIC	LMR	BLRT	Entropy	Class probability (%)
1	6953.503	6976.563	6935.538				1
2	6765.124	6734.276	6746.725	0.000	0.000	0.860	0.37/0.63
3	6602.354	6663.363	6652.431	0.004	0.000	0.887	0.28/0.34/0.38
4	6515.775	6568.658	6506.273	0.085	0.000	0.896	0.26/0.23/0.20/0.31
5	6406.034	6370.138	6362.185	0.191	0.000	0.904	0.16/0.24/0.21/0.09/0.30

### The average attribution rate and naming of the three categories of volume overload management behavioral ability trajectory

According to the LCGM model, combined with the characteristics of the change trajectory of the volume management ability of peritoneal dialysis patients, the volume management ability was divided into three subgroups. The average probabilities of each category of PD patients belonging to each latent category were 0.974, 0.972, and 0.980, respectively (Table 3).

**Table 3:** Average assigned volume rate of three types of volume overload management behavioral ability trajectory (*n* = 233).

Model	C1	C2	C3
C1	0.974	0.018	0.008
C2	0.016	0.972	0.012
C3	0.010	0.002	0.980

The C1 group consisted of 79 patients, accounting for 33.9% of the overall level. In the initial 6-month period of dialysis, the volume overload management ability of patients gradually improved from a low level. Therefore, the C1 group is named the “low-rise group.”

The C2 group included 88 patients, accounting for 37.8% of the total. The volume-load management ability of patients gradually increased from a moderate level. So, it is named the “medium-level increase group.”

**Figure 1:**
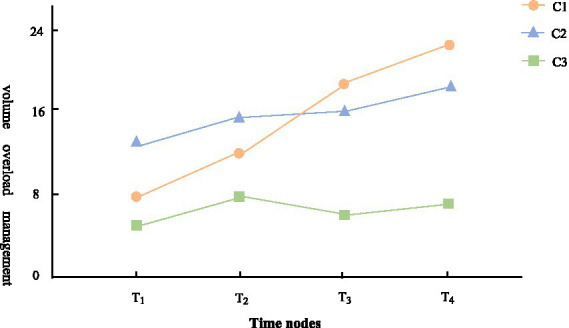
The latent class growth model trajectory of volume-load management behavior of peritoneal dialysis patients.

The C3 group consisted of 66 patients, accounting for 28.3% of the total. The volume-load management ability of patients fluctuates around the low–medium level. Thus, the C3 group is named the “low–medium level fluctuation group.” The specific trends are shown in Figure 1.

### Influencing factors of the change trajectory of volume management behavior ability in peritoneal dialysis patients

The general data of peritoneal dialysis patients in the three latent categories were compared. The results indicated that there were statistically significant differences in the grouping of changes in the behavioral ability of volume-load management among peritoneal dialysis patients in terms of educational level (*χ*^2^ = 15.344, *p* = 0.018), diabetic nephropathy (*χ*^2^ = 11.267, *p* = 0.004), concurrent peritonitis during this period (*χ*^2^ = 11.340, *p* = 0.003), and hypoproteinemia (*χ*^2^ = 7.700, *p* = 0.021), as presented in Table 4.

**Table 4:** Analysis results of influencing factors of three latent categories of volume-load management behavior of peritoneal dialysis patients.

Variables	C1 (*n* = 79)	C2 (*n* = 88)	C3 (*n* = 66)	*χ* ^2^	*p*
Age (years)					
<45	17	22	10	3.286	0.511
45–59	41	47	35		
≥60	21	19	21		
Sex					
Male subjects	47	48	40		
Female subjects	32	40	26		
Marital status					
Married	73	81	52	8.442	0.077
Unmarried	2	3	5		
Divorced	4	4	9		
Place of residence					
Rural	26	27	24	0.551	0.759
Towns	53	61	42		
Level of education					
Primary school and below	15	13	18	15.344	0.018
Junior high school	27	22	26		
Senior high school	22	29	18		
College and above	15	24	4		
Income level (yuan)					
<2,000	14	13	12	1.049	0.984
2,000–4,000	25	26	21		
4,001–6,000	24	31	22		
>6,000	16	18	11		
Occupations					
Enterprises and institutions	8	11	3	5.459	0.709
Farmers	11	15	12		
Staff/clerks	31	35	24		
Retirement	14	17	15		
No regular occupation	15	10	12		
Hypertension					
Yes	55	53	41	1.726	0.422
No	24	35	25		
Diabetic nephropathy					
Yes	39	32	42	11.267	0.004
No	40	56	24		
Coronary heart disease					
Yes	31	29	25	0.882	0.643
No	48	59	41		
Concurrent peritonitis during this period					
Yes	16	15	26	11.340	0.003
No	63	73	40		
Take diuretics					
Yes	35	45	24	3.336	0.189
No	44	43	42		
Hypoproteinemia					
Yes	30	26	34	7.700	0.021
No	49	62	32		
Body weight trends					
< ± 5%	63	71	42	7.107	0.130
±5–±10%	7	8	11		
> ± 10%	9	9	13		

Hypoproteinemia was diagnosed by serum albumin less than 3.5 g/dL, as determined by the blood standard at the time of the patient’s first admission.

### Logistic factor analysis of the change trajectory of volume load management behavior ability in peritoneal dialysis patients

Using group C3 as a reference, the following values were assigned: educational level (primary school and below = 1, junior high school = 2, senior high school = 3, college and above = 4, with primary school and below as the control), diabetic nephropathy (yes = 1, no = 0, with no as the control), concomitant peritonitis (yes = 1, no = 0, with no as the control), and hypoproteinemia (yes = 1, no = 0, with no as the control). Logistic regression analysis showed that education level, diabetic nephropathy, concurrent peritonitis, and hypoproteinemia were the factors influencing the change trajectory of peritoneal dialysis volume load management behavior (*p* < 0.05) (Table 5).

**Table 5:** Logistic factor analysis of change trajectory of volume-load management behavior in peritoneal dialysis patients (*n* = 233).

Items	*B*	SD	Wald’s *χ*^2^	*p*	OR	95% CI
C1 vs. C3						
Constants	2.142	0.402	28.391	<0.001	–	–
Level of education						
Junior high school	−0.353	0.104	11.521	<0.001	0.703	0.573–0.861
High school	−0.290	0.088	10.860	<0.001	0.748	0.630–0.889
College and above	−0.556	0.210	7.010	0.019	0.573	0.380–0.866
Diabetic nephropathy	0.405	0.143	8.021	0.006	1.499	1.133–1.984
Concurrent peritonitis during this period	0.911	0.377	5.839	0.032	2.487	1.188–5.207
Hypoproteinemia	0.676	0.196	11.895	<0.001	1.966	1.339–2.887
C2 vs. C3						
Constants	2.512	0.299	70.582	<0.001	–	–
Level of education						
Junior high school	−0.155	0.048	10.428	<0.001	0.856	0.780–0.941
High school	−0.123	0.043	8.182	0.005	0.884	0.813–0.962
College and above	−0.274	0.084	10.640	<0.001	0.760	0.645–0.896
Diabetic nephropathy	0.338	0.122	7.676	0.013	1.402	1.104–1.781
Concurrent peritonitis during this period	0.919	0.351	6.855	0.022	2.507	1.260–4.988
Hypoproteinemia	0.534	0.184	8.423	0.003	1.706	1.189–2.446

### To analyze the differences in physical activity among peritoneal dialysis patients with different trajectories of volume-load management behavior

The physical activity of peritoneal dialysis patients with different change trajectories of volume-load management behavior was analyzed and compared. The results showed that during the initial 6 months of peritoneal dialysis (T1–T4), the physical activity scores of each group showed an upward trend [C1: (*F* = 107.250, *p* < 0.001); C2: (F = 107.250, *p* < 0.001); C3: (*F* = 30.194, *p* < 0.001)].

At the T1 stage, the physical activity score of the C2 group was significantly higher than that of the C1 and C3 groups (*p* < 0.01).

At the T2 stage, the physical activity score of the C2 group was significantly higher than that of the C3 group (*p* < 0.001), and the physical activity score of the C1 group was significantly higher than that of the C3 group (*p* < 0.01).

At the T3–T4 stage, the physical activity score of the C1 group was significantly higher than that of the C2 and C3 groups (*p* < 0.01), and the physical activity score of the C2 group was significantly higher than that of the C3 group (*p* < 0.001) (Table 6).

**Table 6:** Analysis of differences in physical activity among peritoneal dialysis patients with different trajectories of change in volume-load management behavior.

Variables	Physical activity score				*F*	*p*
	T1	T2	T3	T4		
Group C1	453.15 ± 55.15	517.20 ± 61.99	603.77 ± 70.57	732.39 ± 75.64	107.250	<0.001
Group C2	490.82 ± 63.44	551.28 ± 53.56	567.16 ± 56.52	606.15 ± 62.37	45.383	<0.001
Group C3	434.26 ± 61.27	485.93 ± 52.82	498.34 ± 57.90	523.75 ± 62.36	30.194	<0.001
*F*	5.405	9.330	27.086	40.152	–	–
Compare the results in pairs	C2 > C1,^**^ C2 > C3	C2 > C3,^***^ C1 > C3	C1 > C2,^**^ C1 > C3, ^***^C2 > C3	C1 > C2,^***^ C1 > C3, ^***^C2 > C3	–	–

C1, low-level increased group; C2, medium-level increased group; C3, low-to-medium-level fluctuated group.

## Discussion

Tan et al. (16) and Li et al. (17) have shown that ineffective volume overload management is an important cause of heart failure and death in patients undergoing peritoneal dialysis. Previous studies have emphasized the importance of volume management in dialysis patients, and poor volume management behaviors are closely related to adverse outcomes and lower survival time of patients (18–20).

This study identified three types of change trajectories of volume management behavior ability in peritoneal dialysis patients using a growth mixture model. This indicates that there is population heterogeneity in the volume management ability of peritoneal dialysis patients, which can be attributed to various reasons.

On the one hand, differences in patients’ individual characteristics, such as age and residual renal function, result in disparities in volume metabolism efficiency. Moreover, the frequency of dialysis and selection of dialysate both affect volume overload management. In contrast, patients’ behavioral compliance exhibited significant stratification. Understanding bias due to education level may lead to differences in patients’ awareness of management behavior, ultimately forming different trajectory subgroups. This necessitates clinical medical staff to analyze the specific conditions of patients, comprehensively consider their volume-load management behavior levels, and conduct targeted interventions and management.

This study found that education level, diabetic nephropathy, concurrent peritonitis, and hypoproteinemia exhibited statistically significant differences in the change trajectory of volume load management behavior ability among peritoneal dialysis patients. Several studies have indicated a significant correlation between education level and fluid volume management behavior ability (21, 22). Differences in the educational level directly influence patients’ cognition and execution ability regarding fluid volume management. Patients with a high education level generally possess stronger health literacy, can accurately understand and implement salt restriction and volume intake monitoring, and can manage their own volume by strictly recording intake and output. Ling et al. (23) discovered that patients with diabetic nephropathy were more prone to having a high volume load. On one hand, diabetic autonomic neuropathy can impair cardiovascular reflex function, resulting in decreased baroreceptor sensitivity and difficulty in coping with volume fluctuations through compensatory mechanisms (24). On the other hand, long-term hyperglycemia accelerates the degeneration of peritoneal function, reduces ultrafiltration efficiency, and necessitates frequent adjustment of dialysate concentration or peritoneal retention time, which may lead to inadequate coping of initial peritoneal dialysis patients and impaired volume overload management behavior (25).

Peritonitis during peritoneal dialysis can directly damage peritoneal barrier function, leading to increased capillary leakage and blocked peritoneal lymph return, resulting in volume overload in the short term. Simultaneously, inflammation enhances the ability of the peritoneum to absorb glucose, and the peak of osmolal-driven ultrafiltration disappears in advance. Hypertonic dialysate is needed to maintain ultrafiltration, but it may aggravate residual renal function damage (26). In addition, the dialysis regimen needs to be adjusted during the treatment of peritonitis, and the increased complexity of the operation may lead to incomplete drainage or recurrence of infection. The overlap of acute events and adaptive interventions causes the fluid volume management trajectory of patients with peritonitis to show significant phase deviation (27).

Peritoneal dialysis patients with hypoproteinemia are affected by fluid volume management through the dual mechanisms of decreased colloid osmotic pressure and nutritional imbalance. On the one hand, decreased plasma albumin leads to extravasation of intravascular volume into the interstitial space, causing anasarca, which requires intensive ultrafiltration but may fail due to insufficient residual renal function (28, 29). In contrast, malnutrition reduces the muscle mass and exercise endurance of patients and reduces their compliance with salt and water restriction, which leads to differences in volume-load management behavior.

This study revealed a general upward trend in physical activity among patients during the first 6 months of initiating peritoneal dialysis, which may be attributed to physiological adaptations to dialysis therapy, enhanced patient-clinician collaborative management, and improved fluid balance. In the first week of dialysis (T1), the medium-level increasing group showed significantly higher physical activity scores than the low-level increasing group and the low-medium fluctuating group, possibly due to better early adaptive recovery and relatively higher initial capacity for fluid load management, reflecting overall better baseline patient conditioning. By the first month (T2), the medium-level increasing group continued to score significantly higher than the low-medium fluctuating group, whereas the low-level increasing group surpassed the latter. These differences may stem from more precise fluid management, such as higher ultrafiltration attainment rates and better preservation of residual renal function, which effectively reduces fatigue associated with fluid overload (30). Effective fluid management helps maintain a stable extracellular volume, optimizes tissue perfusion and oxygen delivery, and directly enhances muscular endurance and functional capacity, thereby providing a physiological foundation for increased physical activity (31). The medium-level increasing group demonstrated stronger treatment adherence early, facilitating fluid balance and muscular recovery, whereas the low-medium fluctuating group likely experienced delayed or suboptimal volume adjustment, leading to tissue edema and impaired energy metabolism that restricted mobility. Between three and 6 months (T3–T4), the low-level increasing group exhibited significantly higher physical activity than both the medium-level increasing group and the low-medium fluctuating group, whereas the medium-level increasing group remained higher than the low-medium fluctuating group. This divergence may reflect the long-term effects of fluid management and differences in dynamic adjustment capacity. Successful fluid management reduces cardiac preload and minimizes interstitial edema, thereby alleviating activity-related dyspnea and bodily heaviness and enabling patients to engage in daily activities with greater ability and confidence (32). The medium-level increasing group benefited from higher ultrafiltration rates and better-preserved residual renal function, effectively controlling fluid retention and improving energy metabolism, thereby supporting physical recovery. However, some patients in this group experienced a gradual decline in peritoneal transport characteristics, requiring frequent adjustments to the dialysate concentration. Delays in such adjustments could lead to fluctuations in ultrafiltration efficiency, thereby limiting further improvement in physical activity. This underscores the importance of dynamic and precise fluid management to sustain physical activity capacity over time.

This study has several limitations. Due to constraints in human resources, the sample size was relatively small, which may have resulted in limited statistical power. Convenience sampling may restrict the generalizability of the findings. Future studies involving multicenter recruitment and larger sample sizes are required to enhance the robustness and applicability of our findings. Furthermore, this investigation only assessed the fluid load management behavior ability in peritoneal dialysis patients during the initial 6 months of dialysis; longer-term longitudinal studies are needed to observe the evolution of this capacity over an extended period. Finally, when incorporating factors influencing different trajectories of fluid load management behavior, potential omissions, such as bioimpedance and NT-proBNP, might have occurred. Subsequent studies should address these limitations to refine the findings.

## Conclusion

The behavioral ability of volume load management in peritoneal dialysis patients within the first 6 months of dialysis presents three trajectories. There were differences in the curve trajectories of volume-load management behavior among the patients. Education level, diabetic nephropathy, concurrent peritonitis, and hypoproteinemia influenced the change in trajectories. Volume-load management ability at different time stages affects the physical activity of patients. Enhancing the volume-load management ability of patients at different stages is conducive to improving their physical activity and has a positive impact on enhancing their motor function and prognosis.

## Data Availability

The original contributions presented in the study are included in the article/supplementary material, further inquiries can be directed to the corresponding author.
